# Correlation of Controlling Nutritional Status Score and Systemic Immune‐Inflammation Index With Frailty and Prognosis in Chronic Heart Failure Patients With Atrial Fibrillation

**DOI:** 10.1155/crp/5244972

**Published:** 2026-06-21

**Authors:** Jun Wang, Jun Wu Xing, Guang Gan Bo

**Affiliations:** ^1^ Department of Cardiology, Nanjing Integrated Traditional Chinese and Western Medicine Hospital Affiliated with Nanjing University of Chinese Medicine, Nanjing, 210014, Jiangsu, China

**Keywords:** atrial fibrillation, chronic heart failure, controlling nutritional status score, frailty, prognosis, systemic immune-inflammation index

## Abstract

**Objective:**

To explore the predictive value of the controlling nutritional status (CONUT) score combined with the systemic immune‐inflammation index (SII) for frailty and prognosis in chronic heart failure (CHF) patients with atrial fibrillation (AF).

**Methods:**

This retrospective study included 264 patients with CHF and AF who were admitted to and treated at the inpatient and outpatient departments of Nanjing Integrated Traditional Chinese and Western Medicine Hospital from January 2016 to July 2019. Follow‐up ended upon patient death, with the final follow‐up deadline set for September 2024. Binary logistic regression and Cox regression were used to identify factors influencing frailty and all‐cause mortality. The cutoff values for the CONUT score and SII are determined by the ROC diagnostic threshold for mortality. Cutoff values for the CONUT score and SII were 6.5 and 990.2, respectively, categorizing patients into four groups: G1 (high CONUT ≥ 6.5 and high SII ≥ 990.2), G2 (high CONUT ≥ 6.5 and low SII < 990.2), G3 (low CONUT < 6.5 and high SII ≥ 990.2), and G4 (low CONUT < 6.5 and low SII < 990.2).

**Results:**

Frailty was present in 165 patients (165/264). Multivariate logistic regression identified disease duration, CONUT score, and SII as significant factors for frailty. A positive correlation between CONUT and SII was observed (*r* = 0.648, *p* < 0.001). Cox regression analysis found smoking, CONUT, and SII as risk factors for mortality. Survival analysis showed lower survival rates in high CONUT and high SII groups (*p* < 0.001), with the G1 group having the lowest survival rate compared to G2, G3, and G4 (*p* < 0.001).

**Conclusion:**

The CONUT score and SII are useful predictors of frailty and mortality in CHF patients with AF. Their combined use may improve the assessment of frailty and prognosis in this population.

## 1. Introduction

Chronic heart failure (CHF) is a clinical syndrome characterized by abnormal cardiac structure and/or function, resulting in increased intracardiac pressure or insufficient cardiac stroke volume at rest or during exercise. Atrial fibrillation (AF) is a common arrhythmia that significantly contributes to the development of CHF and increases the risk of major adverse cardiovascular events (MACE) in these patients [1, 2]. Frailty refers to a state of reduced physiological reserve in the elderly, leading to increased vulnerability and decreased ability to withstand stress [3]. Studies indicate that frailty occurs in 15%–74% of CHF patients and in 5.9%–89.5% of AF patients [4, 5]. Therefore, the prevalence and characteristics of frailty in patients with comorbid CHF and AF warrant further clinical investigation. Frailty is associated with higher mortality and hospitalization rates in the elderly, and its presence in CHF patients with AF often signals a poor prognosis, highlighting its clinical importance [6, 7].

Both malnutrition and inflammation play critical roles in the development of CHF with AF and frailty. The incidence of malnutrition in CHF patients ranges from 20% to 70%, with cachexia affecting 15% of patients [7, 8]. Nutritional status has been shown to influence the onset of arrhythmias [9, 10]. Inflammation is also a key factor in myocardial fibrosis and the progression of CHF with AF [11]. Additionally, nutritional status is closely linked to frailty [12], and emerging research has shown that frailty is closely associated with immune and inflammatory responses, giving rise to the concepts of “immune frailty” and “inflammatory frailty” [13, 14].

The controlling nutritional status (CONUT) score assesses a patient’s nutritional status based on their daily intake relative to metabolic needs. Studies have demonstrated that the CONUT score is associated with in‐hospital mortality and infection rates in acute heart failure patients [15]. Studies have demonstrated that three nutritional assessment scores—the Geriatric Nutritional Risk Index (GNRI), the CONUT score, and the Prognostic Nutritional Index (PNI)—are all significant risk factors for adverse prognosis in patients with nonvalvular AF [16]. The systemic immune‐inflammation index (SII) is a reliable indicator of both local and systemic immune responses and is closely linked to CHF development [17]. This study investigates the correlation between the CONUT score and SII with frailty and prognosis in patients with CHF and AF.

## 2. Materials and Methods

### 2.1. General Information

This retrospective study included 264 patients with CHF and AF who were admitted to and treated at the inpatient and outpatient departments of Nanjing Integrated Traditional Chinese and Western Medicine Hospital from January 2016 to July 2019. Including patient gender, age, body mass index, disease course, smoking history, hypertension history, and diabetes history. Ethical approval for this study was obtained from the Ethics Committee of Nanjing Integrated Traditional Chinese and Western Medicine Hospital (Approval No: KYLS‐2025003). The study was registered at the National Clinical Trial Registration Center.

### 2.2. Inclusion Criteria

(1) Diagnosis of CHF and AF according to established criteria [18];
(2) Completion of a frailty score assessment before treatment;
(3) Completion of CONUT score and SII component testing before treatment;
(4) Availability of complete clinical and follow‐up data.

### 2.3. Exclusion Criteria

(1) Diagnosis of malignant tumors;
(2) Previous or current chemotherapy treatment;
(3) Abnormal coagulation or bone marrow function;
(4) Acute or chronic infectious diseases;
(5) Hemorrhagic diseases;
(6) Recent or ongoing antibiotic treatment;
(7) Autoimmune diseases.

**FIGURE 1:**
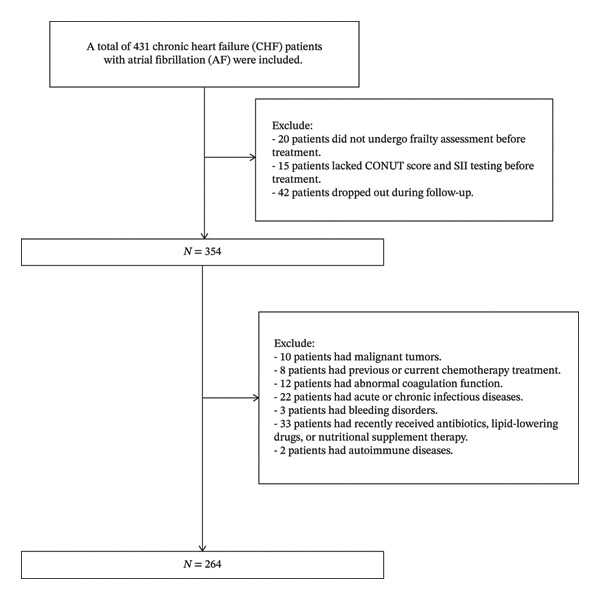
Flowchart.

The aforementioned exclusion criteria were established because these conditions could potentially exacerbate frailty or confound the observation of the indicators CONUT and SII, as shown in Figure 1.

### 2.4. Data Extraction

Clinical data for the included patients were extracted from the inpatient and outpatient systems of the hospital. Relevant data included lymphocyte CONUT, serum albumin levels, total cholesterol levels at admission, and follow‐up information for statistical analysis.

### 2.5. Methods

Frailty was assessed using the Chinese version of the Frailty Scale (Tilburg Frailty Indicator [TFI]). The total score ranges from 0 to 15, with a score > 5 indicating frailty. Blood samples were collected from patients at 8 a.m. on the day of admission to measure the indicators related to the CONUT score and SII. The CONUT score is based on three parameters: serum albumin, total lymphocyte CONUT, and total cholesterol level. The scoring criteria are as follows:

Serum albumin: ≥ 35 g/L (0 points), 30–34.9 g/L (2 points), 25–29.9 g/L (4 points), < 25 g/L (6 points);

Total lymphocyte CONUT: ≥ 1.60 × 10^9^/L (0 points), 1.20–1.59 × 10^9^/L (1 point), 0.8–1.19 × 10^9^/L (2 points), < 0.8 × 10^9/L (3 points);

Total cholesterol: ≥ 180 mg/dL (0 points), 140–179 mg/dL (1 point), 100–139 mg/dL (2 points), and < 100 mg/dL (3 points).

The CONUT score is the sum of the scores for these three components [18]. A score of 0–1 is considered normal, 2–4 indicates mild malnutrition, 5–8 indicates moderate malnutrition, and 9–12 indicates severe malnutrition. The SII is calculated as: SII = platelet CONUT (× 10^9^/L) × neutrophil CONUT (× 10^9^/L)/lymphocyte CONUT (× 10^9^/L). The CONUT score reflects the nutritional status of the patient [19], and the SII serves as an indicator of systemic inflammation [17].

All patients were followed for 5 years via phone or outpatient visits. Those with stable conditions were routinely seen in the outpatient department every 6 months. For patients who missed visits, follow‐up was conducted by phone with the patients or their family members to inquire about their status. Follow‐up ended upon patient death, with the final follow‐up deadline set for September 2024. Total survival time (OS) was recorded for all patients.

### 2.6. Grouping

Patients were categorized into two groups based on the presence of frailty: the frailty group (165 cases) and the nonfrailty group (99 cases). Based on survival status, patients were further divided into the survival group (196 cases) and the death group (68 cases). For analysis of CONUT score and SII levels, cutoff values for predicting mortality in CHF combined with AF were used to divide patients into four subgroups:

 G1: 40 patients with high CONUT score and high SII;
 G2: 12 patients with high CONUT score and low SII;
 G3: 85 patients with low CONUT score and high SII;
 G4: 127 patients with low CONUT score and low SII.

### 2.7. Observation Indicators

The observation indicators were as follows: (1) to compare the factors influencing frailty in patients with CHF and AF; (2) to assess the impact of frailty on the prognosis of patients with CHF and AF; (3) to examine the correlation between CONUT score and SII; (4) to analyze the factors influencing mortality in patients with CHF and AF using Cox regression; (5) to evaluate the predictive value of CONUT score and SII for mortality in patients with CHF and AF; and (6) to assess the impact of different subgroups on the survival time of patients with CHF and AF.

### 2.8. Statistical Analysis

Data were analyzed using SPSS 22.0 statistical software. Continuous data that follow a normal distribution are presented as mean ± standard deviation (‾X ± SD), while those that do not follow a normal distribution are presented as medians (P25, P75). Independent sample *t*‐tests were used for continuous variables with normal distribution. For non‐normally distributed data, the Mann–Whitney *U* test was applied for group comparisons. Pearson’s chi‐square test was used for categorical variables. Variables with statistically significant differences were included in a binary logistic regression model to analyze various factors associated with frailty in patients with congestive heart failure (CHF) complicated by AF. The CONUT score was included to draw the receiver operating characteristic (ROC) curve and calculate the area under the ROC curve (area under the receiver operating characteristic curve, AUROC) to predict frailty. Spearman correlation analysis was used to analyze the correlation between CONUT and SII. In this study, the cutoff values of CONUT and SII were determined by maximizing the Youden index. The Youden index is defined as sensitivity + specificity −1. The Kaplan–Meier method was used to estimate the survival function for each group. The survival probability at each time point was calculated as the product of the conditional probabilities of survival up to that time. The log‐rank test was used to compare the survival distributions between the groups. The null hypothesis of the log‐rank test is that there is no difference in the survival functions between the groups. Multivariate Cox regression was used to assess the relationship between SII, CONUT score, and the prognosis of patients with CHF and AF. In the process of constructing the multivariate model, to ensure the reliability and accuracy of the model, it is recommended to report variance inflation factor (VIF) values for variables included in the multivariate model to exclude multicollinearity between variables. A *p* value of < 0.05 was considered statistically significant.

## 3. Results

### 3.1. Comparison of General Information

Among the patients with CHF and AF, 165 were in the frailty group, and 99 were in the nonfrailty Group. A comparison of general characteristics revealed that the age, CHF with AF duration, N‐terminal pro‐B‐type natriuretic peptide (NT‐proBNP), CONUT score, and SII in the frailty group were significantly higher than those in the nonfrailty group (all *p* < 0.05), as shown in Table 1.

**TABLE 1:** Comparison of general information.

Categories	Frailty group (*n* = 165)	Nonfrailty group (*n* = 99)	*χ*2/t/Z value	*p*
Gender (male:female)	101:64	61:38	0.004	0.948
Age (years)	65.9 ± 13.1	59.8 ± 10.9	4.098	< 0.001
Body mass index (BMI) (kg/m2)	22.57 ± 2.84	23.14 ± 2.37	1.259	0.210
CHF with AF duration (years)	6.3 ± 1.6	4.7 ± 1.8	7.281	< 0.001
Smoking history [*n* (%)]	109 (66.06)	57 (57.58)	1.908	0.167
Hypertension	133 (80.61)	77 (77.78)	0.304	0.581
Diabetes	40 (24.24)	20 (20.20)	0.575	0.448
Grade II (NYHA class)	40 (24.24)	20 (20.20)	0.513	0.474
Grades III–IV (NYHA class)	125 (75.76)	79 (79.80)		
Hemoglobin (g/L)	113.4 ± 14.8	116.1 ± 15.3	1.379	0.169
Serum creatinine (µmol/L)	84.8 ± 17.8	81.0 ± 17.4	0.552	0.582
Urea nitrogen (mmol/L)	5.81 ± 1.27	5.80 ± 0.97	0.073	0.942
Blood uric acid (mmol/L)	398.6 ± 73.2	387.0 ± 74.0	0.463	0.216
Serum sodium (mmol/L)	135.4 ± 8.1	134.6 ± 8.0	0.759	0.448
Serum potassium (mmol/L)	4.58 ± 0.75	4.54 ± 0.71	0.356	0.722
Prothrombin time (PT) (s)	16.62 ± 4.67	15.57 ± 5.07	1.716	0.087
Activated partial thromboplastin time (APTT) (s)	28.78 ± 2.80	28.67 ± 2.63	0.286	0.775
Left ventricular ejection fraction (LVEF) (%)	40.03 ± 3.35	40.89 ± 4.45	1.646	0.102
Left ventricular end‐diastolic diameter (LVEDD) (mm)	56.89 ± 4.46	57.16 ± 4.39	0.481	0.631
NT‐proBNP (pg/mL)	2730.11 (1788.00, 3480.00)	2455.92 (2376.00, 3160.00)	2.204	0.029
CONUT score (points)	5.09 ± 2.37	3.12 ± 2.20	6.721	< 0.001
SII	1193.20 (782.84, 1630.16)	680.84 (611.44, 744.43)	7.809	< 0.001

*Note:* *χ* ^2^ is chi‐square test, *t* is *t*‐test, and *Z* is rank sum test.

### 3.2. Logistic Regression Analysis of Factors Influencing Frailty in Patients With CHF and

AF, frailty status (1 = *frailty present*, 0 = *frailty absent*), was used as the dependent variable. Multivariate logistic regression was conducted, including variables that showed significant differences in univariate analysis. CHF with AF duration [OR (95% CI): 3.329 (1.906–5.504), *p* < 0.001], NT‐proBNP [OR (95% CI): 1.001 (1.000–1.001), *p* = 0.001], CONUT score [OR (95% CI): 1.225 (1.040–1.443), *p* = 0.015], and SII [OR (95% CI): 1.001 (1.001–1.002), *p* = 0.001] were identified as significant factors for the occurrence of frailty in patients with CHF and AF, as shown in Table 2.

**TABLE 2:** Logistic regression analysis of factors influencing frailty in patients with CHF and AF.

Variable	*B*	SE	Wald	OR value (95% CI)	*p*
Constant	−5.238	1.046	25.934	—	< 0.001
Age	0.022	0.013	2.621	1.022 (0.995–1.049)	0.105
CHF with AF duration	1.054	0.220	22.931	2.868 (1.863–4.415)	< 0.001
NT‐proBNP	0.001	0.000	11.865	1.001 (1.000–1.001)	0.001
CONUT score	0.189	0.089	4.509	1.208 (1.015–1.438)	0.034
SII	0.001	0.000	7.294	1.001 (1.001–1.002)	0.005

### 3.3. Correlation Analysis of CONUT Score and SII

**FIGURE 2:**
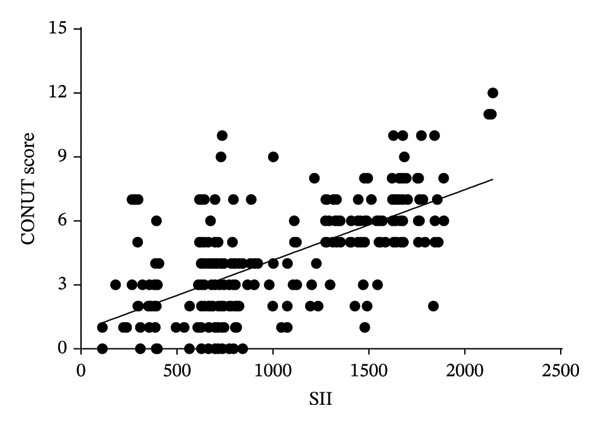
Correlation analysis of CONUT score and SII.

A correlation analysis showed a positive association between the CONUT score and SII (*r* = 0.628, *p* < 0.001), as illustrated in Figure 2.

### 3.4. Comparison of 5‐Year Mortality Rates

**FIGURE 3:**
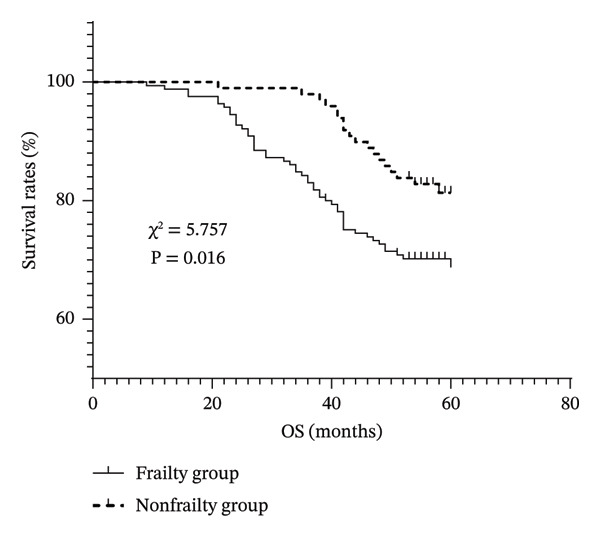
Comparison of 5‐year total survival time.

The 5‐year mortality rate in the frailty group was 30.30% (50/165), while the mortality rate in the nonfrailty group was 18.18% (18/99). Statistical comparison showed a significant difference (*p* < 0.05). The average total survival time in the frailty group was 52.0 months, significantly shorter than the 57.0 months observed in the nonfrailty group (*χ* ^2^ = 5.757, *p* = 0.016) (HR: 1.907, 95% CI: 1.175–3.093), as shown in Figure 3.

### 3.5. Comparison of General Information of Patients With Different Survival Outcomes

Among the included patients, 68 cases resulted in death. A comparison of the general characteristics between the death and survival groups revealed that the proportion of age, CHF with AF duration, LVEF (%), smoking history, CONUT score, and SII were significantly higher in the death group, while LVEF was lower than in the survival group (all *p* < 0.05), as shown in Table 3.

**TABLE 3:** Comparison of general information of patients with different survival outcomes.

Categories	Mortality group (*n* = 68)	Survival group (*n* = 196)	*χ* ^2^/t/Z value	*p*
Gender (male:female)	45:23	117:79	0.895	0.334
Age (years)	69.2 ± 12.9	61.7 ± 12.04	4.201	< 0.001
BMI (kg/m^2^)	23.48 ± 1.98	23.24 ± 2.73	0.771	0.442
CHF with AF duration (years)	6.2 ± 1.6	5.5 ± 1.9	2.705	0.008
Smoking history [*n* (%)]	35 (51.47)	63 (32.14)	8.080	0.004
Hypertension	57 (83.82)	153 (78.06)	1.030	0.310
Diabetes	13 (19.11)	47 (23.98)	0.680	0.410
Grade II (NYHA class)	13 (19.12)	47 (23.98)	0.680	0.410
Grades III–IV (NYHA class)	55 (80.88)	149 (76.02)		
Hemoglobin (g/L)	117.2 ± 12.6	113.4 ± 15.7	1.872	0.076
Serum creatinine (µmol/L)	81.1 ± 18.4	83.0 ± 17.3	1.155	0.249
Urea nitrogen (mmol/L)	5.89 ± 1.04	5.77 ± 1.20	0.743	0.458
Blood uric acid (mmol/L)	399.6 ± 76.7	392.4 ± 72.6	0.689	0.491
Serum sodium (mmol/L)	135.1 ± 7.6	135.0 ± 8.3	0.075	0.940
Serum potassium (mmol/L)	4.59 ± 0.77	4.56 ± 0.72	0.326	0.745
PT (s)	16.69 ± 3.69	16.06 ± 5.18	1.084	0.280
APTT (s)	28.03 ± 2.80	28.58 ± 2.68	1.837	0.120
LVEF (%)	38.34 ± 3.48	40.06 ± 3.69	5.314	< 0.001
LVEDD (mm)	57.26 ± 4.57	56.90 ± 4.38	0.588	0.557
NT‐proBNP (pg/mL)	2533.11 (1868.00, 3364.00)	2659.94 (1705.00, 3370.00)	0.784	0.378
CONUT score (points)	6.23 ± 2.70	3.70 ± 2.15	8.076	< 0.001
SII	1354.10 (1087.59, 1674.41)	928.35 (680.17, 1390.68)	6.641	< 0.001

*Note:* *χ* ^2^ is chi‐square test, *t* is *t*‐test, and *Z* is rank sum test.

### 3.6. COX Regression Analysis of Factors Influencing Mortality in Patients With CHF and AF

COX multivariate regression analysis identified age, smoking, CONUT score, and SII as significant risk factors for death in patients with CHF and AF; LVEF is a protective factor in patients with CHF and AF (Table 4).

**TABLE 4:** COX regression analysis of factors influencing mortality in patients with CHF and AF.

Variables	*β*	SE	Wald value	OR value (95% CI)	*p*
Age	0.036	0.014	6.108	1.039 (1.012–1.067)	0.013
CHF with AF duration	0.011	0.100	0.013	1.012 (0.831–1.231)	0.909
Smoking	0.777	0.356	4.762	2.174 (1.082–4.367)	0.029
LVEF (%)	−0.182	0.047	14.975	0.833 (0.760–0.914)	< 0.001
CONUT score	0.419	0.098	18.123	1.520 (1.254–1.844)	< 0.001
SII	0.001	0.000	9.584	1.001 (1.000–1.001)	0.002

### 3.7. Diagnostic Value of CONUT Score and SII for Mortality in Patients With CHF and AF

**FIGURE 4:**
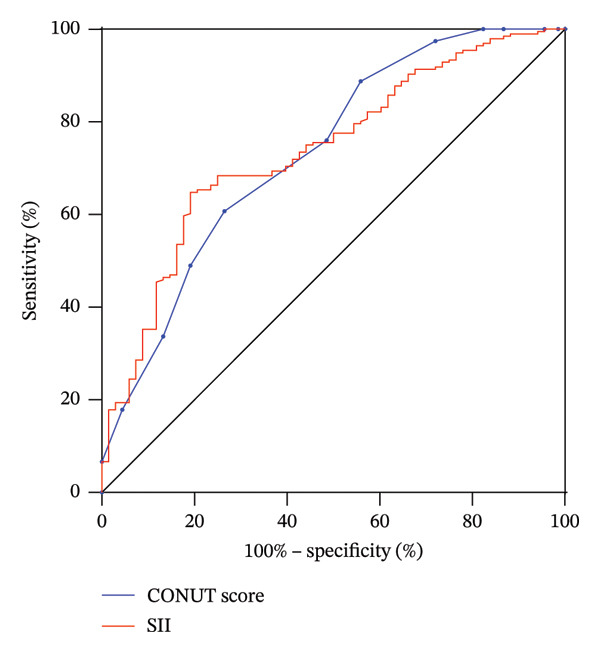
ROC curve for CONUT score and SII in predicting mortality in patients with CHF and AF.

The area under the ROC curve (AUC) for the CONUT score in predicting death in patients with CHF and AF was 0.733. At a cutoff value of 6.5, the Youden index was 0.329, with a specificity of 0.888 and sensitivity of 0.441. For the SII, the AUC was 0.738. At a cutoff value of 990.2, the Youden index was 0.457, with a specificity of 0.648 and sensitivity of 0.809, as shown in Figure 4.

### 3.8. Correlation Between Different Levels of CONUT Score and SII and Prognosis of Patients With CHF and AF

**FIGURE 5:**
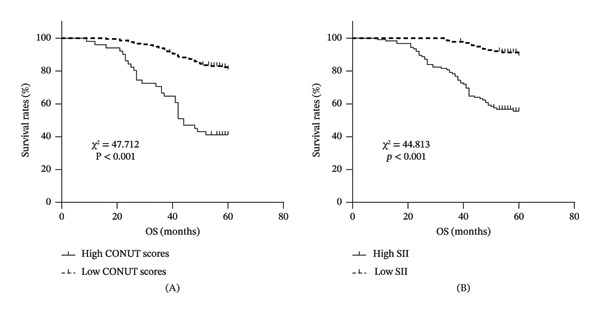
Correlation between different levels of CONUT score and SII expression and prognosis. (A) Survival curve for different CONUT score expression levels. (B) Survival curve for different SII expression levels.

Using the cutoff values for the CONUT score (6.5) and SII (990.2) derived from the ROC analysis, patients were categorized into high and low expression groups for both variables. There were 52 cases in the high‐expression group of the CONUT score, 212 in the low‐expression group, 125 in the high‐expression group of SII, and 105 in the low‐expression group. Survival analysis showed that patients with high CONUT scores had a lower survival rate than those with low CONUT scores (*χ* ^2^ = 47.712, *p* < 0.001) (OR: 4.593, 95% CI: 2.346–8.993), and similarly, patients with high SII expression had a lower survival rate compared to those with low SII expression (*χ* ^2^ = 44.813, *p* < 0.001) (OR: 6.042, 95% CI: 3.728–9.793), as shown in Figure 5.

### 3.9. Correlation Between Different Subgroups and Prognosis in Patients With CHF and AF

**FIGURE 6:**
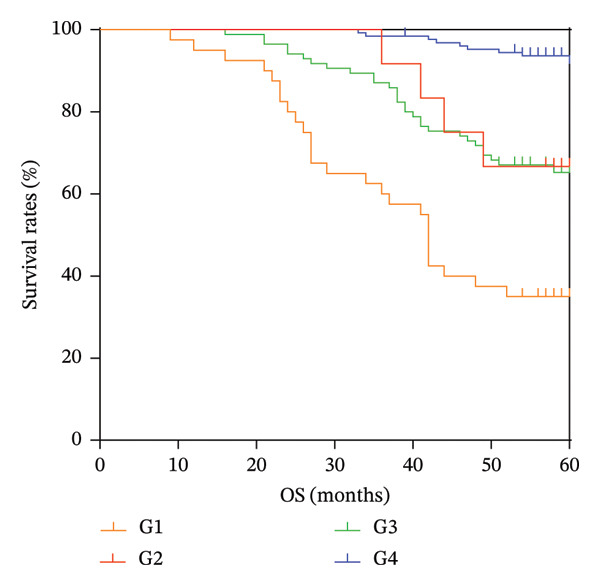
Survival curve analysis of different subgroups and all‐cause mortality in patients with CHF and AF.

The study included 40 cases in G1 (mortality rate: 27/40), 12 cases in G2 (mortality rate: 4/12), 85 cases in G3 (mortality rate: 29/85), and 127 cases in G4 (mortality rate: 9/127). Survival analysis showed that the survival rate in G1 was significantly lower than that in G2, G3, and G4 (*p* < 0.001), while the survival rate in G1 was higher than that in G2, G3, and G4 (*p* < 0.05), as shown in Figure 6.

## 4. Discussion

This study further identified disease duration, the CONUT score, and SII as key risk factors for frailty in patients with CHF complicated by AF. Progressive cardiac functional decline throughout the disease course ultimately contributes to poor prognosis. Our analysis revealed a significant positive correlation between the CONUT score and SII. This suggests that worsening nutritional status (reflected by higher CONUT scores) is associated with an intensified systemic inflammatory response (indicated by higher SII), potentially creating a vicious cycle where inflammation further depletes nutritional reserves. Survival analysis demonstrated a significantly higher mortality rate in the frailty group compared to the nonfrailty group, with 68 deaths occurring during the 5‐year follow‐up period. Multivariate Cox regression analysis of deceased patients confirmed that increased smoking, a high CONUT score, and a high SII were significant independent risk factors for mortality in this CHF‐AF cohort. The association between elevated SII and mortality in CHF patients is well‐established. To further elucidate the combined impact of inflammation and nutrition, patients were stratified based on CONUT scores and SII levels. This stratification revealed that patients with concurrent high CONUT scores and high SII levels (Group G1) exhibited the lowest survival rate and the poorest prognosis. Conversely, patients with both low CONUT scores and low SII levels (Group G4) demonstrated the highest survival rate and the most favorable prognosis. These findings underscore that both the CONUT score (reflecting nutritional status) and SII (reflecting systemic inflammation) are closely and independently associated with mortality risk. The co‐occurrence of malnutrition and inflammation observed aligns with established pathophysiological links.

AF is the most common arrhythmia in patients with CHF. Previous studies have shown that 33% of CHF patients also have AF [20], which is associated with common pathogenic factors such as systemic inflammation induced by conditions like hypertension, diabetes, and obesity [21]. Furthermore, CHF and AF can interact to create a vicious cycle. In patients with CHF, a decline in cardiac diastolic function leads to increased left ventricular end‐diastolic pressure and left atrial pressure, resulting in myocardial remodeling of the left atrium. CHF also activates the renin–angiotensin–aldosterone system, releasing inflammatory factors into the bloodstream and promoting left atrial fibrosis. Once left atrial fibrosis occurs, cell coupling is disrupted, causing low voltage and slow conduction in the left atrium, thereby facilitating the development of AF [22]. The occurrence of AF can damage left ventricular systolic function due to irregular and rapid ventricular rates, leading to increased left ventricular end‐diastolic pressure and reduced cardiac output. AF can also promote left ventricular fibrosis, further inducing CHF [21].

As the population ages, the incidence of frailty increases. A study involving 493,737 individuals reported that the prevalence of frailty was 3% [23]. The risk factors for CHF and AF overlap with those for frailty, making patients with CHF combined with AF more susceptible to frailty. Currently, no studies have reported the incidence of frailty in patients with CHF and AF. This study found that the incidence of frailty in patients with CHF and AF is 62.5%. Frailty is associated with poor prognosis [6], and the high prevalence of frailty in these patients warrants clinical attention.

As the disease progresses, cardiac function continues to decline, ultimately leading to poor prognosis. Studies have indicated that patients with newly diagnosed heart failure have better prognoses compared to those with acute decompensated CHF [24]. Moreover, prolonged disease duration in CHF patients is associated with continuous decline in cardiac function and poor prognosis [25]. As CHF cardiac function deteriorates, the incidence of frailty also increases [26]. The CONUT score and SII reflect nutritional and systemic inflammatory factors. The CONUT score, which combines serum albumin, lymphocyte CONUT, and total cholesterol, is unaffected by subjective patient factors or fluid retention, providing a more accurate measure of nutritional immune status and energy expenditure in CHF patients [27]. While no studies have explored the correlation between CONUT score and AF, hypoproteinemia, reflected in the CONUT score, has been linked to frailty in patients [28]. Lymphocytes, essential for immune regulation, decline with age, contributing to immune aging and promoting organ inflammation [29]. Chronic inflammation is a potential mechanism for frailty, and the neutrophil‐to‐lymphocyte ratio, a marker of inflammation, may indicate frailty’s onset and progression [30]. Total cholesterol is an indicator of lipid status. Research has shown that higher cholesterol levels are associated with a lower incidence of frailty [31]. A study involving descendants of long‐lived individuals suggests that longevity may be linked to elevated total cholesterol levels [32]. Previous studies have shown that the hemoglobin, albumin, lymphocyte, and platelet (HALP) score is an independent risk factor for all‐cause mortality in patients with nonvalvular AF [33]. SII is a marker of systemic inflammation, with chronic inflammation being a significant risk factor for poor prognosis in patients with CHF combined with AF and for the development of frailty [34–36]. Comprising neutrophils, lymphocytes, and platelets, SII reflects inflammatory responses. Inflammation stimulates megakaryocytes to proliferate, increasing platelet production. Elevated platelets interact with endothelial cells and leukocytes, triggering inflammatory responses and creating a vicious cycle. The onset of many diseases is associated with increased platelet levels [37].

This study shows a positive correlation between CONUT score and SII, suggesting that higher CONUT scores reflect worse nutritional status and that an intensified inflammatory response further depletes the body’s nutrients, resulting in a vicious cycle.

A study in Mexico demonstrated that the mortality rate increased with the frailty index, regardless of gender [38]. Other studies have indicated that higher frailty rates are associated with higher mortality rates in patients [39, 40]. This may be due to frailty increasing patient vulnerability to external stimuli and reducing their ability to cope with stress, along with the pathophysiological changes across multiple systems. Such changes not only impair physical function in the elderly but also significantly increase their risk of death [41].

Studies have shown that smoking is linked to left ventricular structure and function, as well as CHF‐related hospitalizations [42]. Additionally, the number of cigarettes smoked per year has a dose–response relationship with heart failure risk. The longer the time since quitting smoking, the lower the relative risk of heart failure. However, the impact of smoking on increasing heart failure risk may persist for decades [43]. Smoking has also been shown to significantly increase the incidence of AF [44], and it raises the risk of all‐cause and cardiovascular mortality, as well as major bleeding in AF patients using anticoagulants [45]. Smoking damages the cardiovascular system through multiple mechanisms, including inflammation, immune responses, blood lipid changes, and increased blood pressure, contributing to common cardiovascular diseases like arrhythmia and heart failure [46].

Studies have confirmed that a high CONUT score is an independent risk factor for all‐cause mortality after discharge in patients with acute heart failure [47]. A CONUT score greater than 5 in CHF patients is significantly associated with all‐cause mortality [48], likely due to the increased risk of infection associated with malnutrition, which results in poor prognosis [49]. Previous studies have also shown that malnutrition increases mortality in AF patients over 75 years old [50]. A cohort‐based study indicated that using the CONUT score to assess the nutritional status of AF patients correlates with their mortality, embolism, and bleeding risk [51].

A domestic study showed that SII is an independent risk factor for death in CHF patients with renal insufficiency [52]. Another study found that higher SII levels at admission in CHF patients are associated with a higher mortality rate and that SII has a greater predictive value for long‐term prognosis than for short‐term outcomes [53]. Composed of neutrophils, lymphocytes, and platelets, SII reflects the body’s inflammatory state. Many studies have shown that inflammatory markers correlate with the prognosis of AF patients [54, 55]. Cytokines like myeloperoxidase secreted by neutrophils contribute to the inflammatory response, leading to atrial fibrosis and increasing the susceptibility to AF [56]. T lymphocytes (T cells) also play a crucial role in the development of AF. A study involving postcardiac surgery patients showed a significantly higher proportion of CD4+CD28 T cells in blood samples from patients with postoperative AF compared to those without AF (11.1% vs. 1.9%) [57]. Platelets are also key in the inflammatory process in AF. They combine with white blood cells to produce specific inflammatory markers [58]. T cells play a pivotal role in modulating inflammatory responses that directly impact CHF progression and prognosis. Activated T cells infiltrate cardiac tissue and secrete proinflammatory cytokines, exacerbating myocardial fibrosis and ventricular remodeling [59]. Specifically, an imbalance in T‐cell subsets—such as increased Th17 cells and reduced regulatory T cells (Tregs)—promotes sustained inflammation, correlating with higher mortality and hospitalization rates in CHF patients [60]. Furthermore, T‐cell exhaustion, characterized by impaired immune checkpoint signaling, contributes to inadequate inflammation resolution and accelerated heart failure deterioration [61]. These mechanisms collectively underscore T cells as critical mediators linking systemic inflammation to adverse CHF outcomes. Therefore, we conclude that high CONUT scores and high SII levels indicate a poor prognosis for patients with CHF combined with AF.

In an inflammatory state, tumor necrosis factor‐α can cause anorexia and reduced protein intake. This not only inhibits protein synthesis but also promotes protein catabolism [62]. Previous studies have indicated that decreased phagocytic function of neutrophils is a key factor in the coexistence of malnutrition and inflammation. Persistent malnutrition and inflammation lead to reduced immunity, increased inflammatory factor release, and further protein degradation, creating a vicious cycle [63]. Malnutrition and inflammation are also associated with an increased incidence of adverse cardiovascular events, resulting in a poorer prognosis [64, 65]. Therefore, we conclude that malnutrition and a more severe inflammatory state suggest a poor prognosis in patients with CHF combined with AF.

CONUT reflects nutritional status and subclinical inflammation, while SII quantifies immune‐inflammation imbalance [66, 67]. Together, they capture the bidirectional “frailty‐inflammatory‐nutritional” axis in patients with CHF combined with AF, where malnutrition exacerbates immune dysfunction and vice versa, amplifying frailty and mortality risk [68]. Unlike the Fried criteria or Clinical Frailty Scale (CFS), CONUT and SII are derived from routine laboratory data, eliminating subjectivity and enabling reproducible, cost‐effective assessment at hospital admission or during outpatient follow‐up [66, 67]. This is critical in CHF, where timely risk stratification guides management decisions. Compared to the FI (which requires ≥ 30 variables), the CONUT‐SII model achieves comparable or superior prognostic accuracy with only 6 laboratory parameters, reducing administrative burden [69]. While conventional tools like the Fried criteria remain foundational for frailty phenotyping, the CONUT‐SII integration offers a complementary, objective, and scalable alternative for patients with CHF combined with AF. Its ability to simultaneously capture nutritional and immune‐inflammatory derangements—key drivers of frailty in HF—may enhance targeted interventions. Future studies should validate this integrated model against prospective cohorts and compare its net reclassification improvement (NRI) with combined tools like the FI or Fried to formally quantify its additive prognostic value.

Therefore, our findings suggest that in patients with CHF and AF, elevated CONUT score and SII serve as indicators of malnutrition and a proinflammatory state. Active assessment of nutritional intake and inflammatory response status is warranted. Prompt, tailored nutritional interventions are recommended to restore nutritional status and mitigate inflammation, thereby positively impacting disease management.

In summary, integrating CONUT and SII bridges the gap between subjective clinical frailty tools and single‐biomarker approaches by objectively quantifying the nutritional‐inflammation axis. This strategy aligns with the biological complexity of CHF frailty and may improve survival risk stratification, though direct comparisons with FFP, FI, or GNRI in large CHF cohorts are needed to validate its superiority.

However, there are some limitations in this study. ① This is a retrospective study, and confounding factors were not excluded. ② The dynamic impact of CONUT score and SII on prognosis was not observed. ③ This study is a single‐center retrospective study and should be further validated by multicenter prospective studies. ④ The sample size is small and requires further expansion to better evaluate the impact of CONUT score and SII on frailty occurrence and prognosis in patients with CHF combined with AF. ⑤ The blood parameters were derived from a single sample per patient. The lack of longitudinal monitoring to account for potential fluctuations in these values may have influenced the results. ⑥ The retrospective nature of the study is an inherent source of potential bias that cannot be fully eliminated. ⑦ The operational definition of frailty investigated is not a universally standardized parameter; it may vary across populations and is subject to some degree of subjective interpretation. ⑧ The study was conducted at a specialized center in China, employing integrated treatments that include traditional Chinese medicine (TCM). Consequently, the findings may have limited applicability to centers practicing Western medicine exclusively or to different ethnic/population groups. ⑨ The analysis did not include a detailed assessment of crucial inflammatory markers, such as specific cytokines (e.g., IL‐6, TNF‐α) and serum C‐reactive protein (CRP), which are highly relevant for evaluating inflammatory status. Their absence is a notable limitation that should be addressed, either by including such data or by explicitly acknowledging this gap.

In conclusion, our study shows that both CONUT score and SII are associated with frailty and poor prognosis in patients with CHF combined with AF. The combination of these two indicators can aid in prognosis assessment.

## Author Contributions

The formulation of the study concept and design: Jun Wang. Data collection: Guang gan Bo. Data analysis and interpretation: Xingjun Wu. Draft manuscript: Jun Wang. Review and revision of the manuscript: Xingjun Wu. Statistical analyses: Jun Wang.

## Funding

This research was funded by the Engineering Research Center for Evaluation and Transformation of Classical Prescriptions in Traditional Chinese Medicine of Jiangsu Province, Project Name: Mechanism of Zhigancao Decoction in improving atrial fibrosis and inflammatory factor levels in rats with atrial fibrillation by mediating SIRT1 to regulate the PI3K‐AKT and MAPK signaling pathways (No. GCZX‐20240109), the Nanjing Administration of Traditional Chinese Medicine, Project Name: Nanjing Traditional Chinese Medicine Youth Talent Training Program (No. ZYQ20053), and the Nanjing Municipal Health Commission, Project Name: Inheritance of Academic Experience of the Sixth Batch of Senior Traditional Chinese Medicine Experts (No. 064102004).

## Consent

The patients/participants [legal guardian/next of kin] provided written informed consent to participate in this study.

## Conflicts of Interest

The authors declare no conflicts of interest.

## Data Availability

The data that support the findings of this study are available from the corresponding author upon reasonable request.
